# The Impact of METTL3 on MDM2 Promotes Podocytes Injury During Diabetic Kidney Disease

**DOI:** 10.1111/jcmm.70627

**Published:** 2025-05-27

**Authors:** Han Wu, Ziyang Yu, Yitian Yang, Zhuoting Han, Qingjun Pan, Ying Chen, Hongyuan Yu, Siman Shen, Li Xu

**Affiliations:** ^1^ Department of Laboratory Medicine The Second Affiliated Hospital of Guangdong Medical University Zhanjiang China; ^2^ School of Medicine Xiamen University Xiamen China; ^3^ Respiratory Medicine The Second Affiliated Hospital of Guangdong Medical University Zhanjiang China; ^4^ Clinical Research and Experimental Center Affiliated Hospital of Guangdong Medical University Zhanjiang China; ^5^ Department of Nephrology The First Hospital of China Medical University Shenyang China; ^6^ Department of Urology The First Hospital of China Medical University Shenyang China; ^7^ Department of Anesthesiology The Second Affiliated Hospital of Guangdong Medical University Zhanjiang Guangdong China

**Keywords:** dedifferentiation, diabetic kidney disease, murine double minute 2, N6‐Methyladenosine, podocytes

## Abstract

N6‐Methyladenosine (m6A) methylation plays a role in various pathological processes, including renal fibrosis and aging. Our previous studies have highlighted abnormal expression of the methyltransferase enzyme, methyltransferase like 3 (METTL3), in aging kidney tissues. This study aims to elucidate the regulatory mechanisms of METTL3 in diabetic kidney disease (DKD) by establishing a conditional METTL3 knockout model. We observed elevated m6A levels in the kidneys of type I diabetic mice and in cultured mouse podocytes exposed to advanced glycation end products (AGEs). These increases were attributed to enhanced METTL3 expression. Significantly, podocyte‐specific METTL3 knockdown mitigated injury in streptozotocin (STZ)‐induced diabetic mice, evidenced by reduced urine albuminuria and renal pathology. We discovered that METTL3 induced abnormal m6A modification of murine double minute 2 (MDM2), which triggered its degradation in an IGF2BP2 (insulin‐like growth factor 2 mRNA‐binding protein 2)‐dependent manner. This modification led to increased MDM2 expression, activating the Notch signalling pathway and inducing podocyte cell cycle arrest under diabetic conditions, which further released inflammatory factors and caused podocyte dedifferentiation. Our findings suggest that targeting m6A modification via METTL3 could be an effective strategy for treating DKD.

## 1 Introduction

Diabetes mellitus currently affects approximately 529 million individuals worldwide, with projections suggesting an increase to about 1310 million by 2050 [1]. Diabetic kidney disease (DKD), a significant microvascular complication of diabetes mellitus, is the leading cause of end‐stage renal disease. DKD poses severe threats to public health and imposes substantial economic burdens on patients [2]. The disease is characterised by various pathological changes in the kidneys, including podocyte loss, glomerular sclerosis, thickening of the glomerular basement membrane, mesangial matrix expansion, interstitial fibrosis, and tubular atrophy [3, 4]. Podocytes are specialised visceral epithelial cells that line the outer surface of the glomerular basement membrane and interdigitate with neighbouring cells to form the slit diaphragm. Since podocytes have limited repair and regeneration capabilities, the degree of podocyte injury is considered to be the main prognostic determinant of end‐stage renal disease [5]. Although standard tight blood glucose control may slow the progression of DKD, the long‐term efficacy of novel treatments requires further evaluation [6]. The lack of effective interventions on preventing podocyte injury demands a better understanding of the key and universal molecules involved in various podocytopathies, which may provide potential diagnostic and therapeutic measures for patients with DKD.

N6‐methyladenosine (m6A) is a widespread and highly conserved RNA modification in mammals, affecting mRNA or non‐coding RNAs at the transcriptional level. This modification is mediated by the interaction of m6A methyltransferases (‘writers’), demethylases (‘erasers’), and binding proteins (‘readers’). m6A methyltransferases, primarily the heterodimer core complex of methyltransferase‐like 3 (METTL3) and methyltransferase‐like 14, along with Wilms' tumour 1‐associating protein as a splicing factor, attach m6A groups to the N6 position of adenosine residues within the ‘RRACH’ consensus sequence. This modification is abundant near RNA termination codons, 3′ UTRs, and internal exons [7]. Key demethylases include fat mass and obesity‐associated protein and AlkB homologue 5, whereas reader proteins such as YTH N6‐methyladenosine RNA‐binding protein 1/2/3, insulin‐like growth factor 2 mRNA‐binding protein 1/2/3 (IGF2BP1/2/3) can bind m6A motifs, significantly influencing RNA function [8, 9, 10]. Dysregulation of m6A modification has been linked to a variety of kidney diseases [11, 12, 13], Particularly, METTL3 is the N6‐adenosine‐methyltransferase complex catalytic subunit (70 kDa). This enzyme is involved in the post‐transcriptional methylation of internal adenosine residues in eukaryotic mRNAs, forming N6‐methyladenosine (m6A), catalysing methylation reactions that have been implicated in kidney disease [12, 14, 15, 16]. METTL3 is reported to be closely associated with kidney diseases, such as renal ischemic reperfusion injury and DKD. However, the role of RNA m6A methylation and its regulatory mechanism remain unclear in podocytopathies.

In this study, we investigate the role of the m6A methyltransferase METTL3 in podocytes during DKD by developing a conditional podocyte‐specific METTL3 gene knockout mouse model. We aim to determine whether METTL3 regulates mouse doubleminute 2 homologue (MDM2) through aberrant m6A modification, involving IGF2BP2‐dependent degradation pathways. This regulatory mechanism potentially leads to podocyte, re‐entry of cell cycles, dedifferentiation, release of inflammatory cytokines, progression of proteinuria and renal pathological damage. These findings could help identify promising therapeutic targets and reveal a novel strategy for treating DKD.

## 2 Materials and Methods

### 2.1 Animal Experimental Design

All protocols for animal experiments were approved by the Research Ethics Committee of China Medical University. Male C57BL/6J mice were acquired from Vital River (Beijing, China) to establish the diabetic mouse model. Mice were intraperitoneally injected with 55 mg/kg of streptozotocin (STZ) or 0.1 mol/L citrate buffer (control) for five consecutive days starting at 8 weeks of age, as previously described [17]. Successful induction of diabetes was confirmed by blood glucose levels exceeding 300 mg/dL 14 days post‐injection.

For targeted gene knockdown, an adeno‐associated virus (AAV9) carrying shRNA targeting MDM2 (shMDM2), under control of the nephrin promoter, was procured from GeneChem Company (Shanghai, China). This construct was injected in situ into the renal pelvis. Mice were assigned to three experimental groups: (1) control group treated with citrate buffer or empty vector, (2) STZ group and (3) STZ group transfected with NPHS‐shMDM2.

The conditional podocyte‐specific METTL3 knockout (KO) model was generated using METTL3 flox/flox mice obtained from GemPharmatech Co. Ltd. (Nanjing, China). These METTL3fl/fl mice were crossed with mice expressing Cre recombinase under the nephrin promoter (Nphs1‐Cre) to produce Nphs1‐Cre METTL3fl/fl (cKO) mice, whereas the METTL fl/fl mice were used as control. Genotyping was performed via PCR before and after the experiments to confirm the specific knockout of METTL3 in podocytes.

### 2.2 Glomerular Isolation

Glomeruli were isolated in control and STZ mice at 16 weeks old. Samples were prepared in duplicate, and each sample contained the glomeruli of two mice. Glomeruli were isolated from kidney cortical tissue with 45% percoll solution, as previously described [18].

### 2.3 Cell Culture and Treatment

Conditionally immortalised mice podocytes were obtained from the Institute of Basic Medical Sciences, Chinese Academy of Medical Sciences. These cells were cultured in RPMI‐1640 medium supplemented with 10% fetal bovine serum, 100 U/mL penicillin, and 100 μg/mL streptomycin at 33°C under a 5% CO2 atmosphere, as previously described [19]. We utilised cells between passages 10 and 15 to ensure they maintained characteristic podocyte functionality and phenotype. After transitioning the culture to 37°C for 7 days to induce differentiation, podocyte maturity was confirmed by the expression of synaptopodin using immunofluorescence staining, confirming the cells retained key podocyte markers, which are crucial for their functionality and relevance to in vivo conditions.

For the advanced glycation end‐products (AGEs) exposure, mature podocytes were treated with bovine serum albumin (BSA) glycated by incubation with 500 mM glucose for 8 weeks at 37°C to form advanced glycation end‐products (AGEs‐BSA). This AGEs‐BSA, purchased from Sigma‐Aldrich (St. Louis, MO, USA, catalogue number G9295), was used at a concentration of 100 μg/mL to expose podocytes for 24 h, mimicking diabetic conditions. Concurrently, podocytes were also treated with Jagged1 (30 μmol/L, PeproTech, Rocky Hill, NJ, USA) to simulate Notch signalling activation. To assess the effects of gene silencing on the podocytes' response to AGEs, specific targeting sequences of METTL3, IGF2BP2 and MDM2 siRNA (Syngentech, China) were transfected using Lipofectamine 3000 (Invitrogen) according to the manufacturer's instructions.

### 2.4 Quantification of m6A Modifications

m6A levels in RNA were quantified using the EpiQuik m6A RNA Methylation Quantification Kit (colorimetric), following the manufacturer's instructions. Total RNA was extracted from kidney tissues and podocytes, and absorbance was measured at 450 nm.

### 2.5 Dot Blot

The RNA sample was boiled and then cooled on ice, and spotted onto a nylon membrane. The membrane was UV cross‐linked for 5 min (or irradiated for 1 h with a replaced UV lamp) to bind the RNA. Then, it was blocked in 5% skimmed milk on a shaker at room temperature for 2 h, incubated with the m6A primary antibody overnight on a shaker at 4°C, and washed three times the next day with TBST solution on a shaker at room temperature, each wash lasting 10 min. After that, the secondary antibody was applied.

### 2.6 Western Blot

Western blot analysis was performed as previously described [20]. The following antibodies were used at these dilutions for western blot analysis: METTL3 (Abcam ab195352, 1:1000), METTL14 (Sigma, HPA038002, 1:1000), FTO (Proteintech, 27226‐1‐AP, 1:1000), synaptopodin (synap) (Sigma‐Aldrich, SAB3500585, 1:500), podocin (Abcam, ab181143, 1:500), caspase 3 (Abcam, ab184787, 1:500), B‐cell lymphoma/leukaemia‐2 (Bcl2) (Abcam, ab182858, 1:1000), MDM2 (Santa Cruz Biotechnology, sc‐965, 1:500), IGF2BP2 (Proteintech, 11,601‐1‐AP, 1:1000), proliferating cell nuclear antigen (PCNA) (Abcam, ab92552, 1:1000), cyclin B1 (Abcam, ab181593, 1:500), cyclin‐dependent kinase inhibitor 1A (P21) (Proteintech, 28,248‐1‐AP for mouse, 1:1000), Notch intracellular domain (NICD) (Abcam, ab52627, 1:500), and hairy and enhancer of split 1 (Hes1) (Abcam, ab71559, 1:500). Antibody validation was confirmed by assessing specific bands in the expected molecular weight range on western blots with known positive and negative controls.

### 2.7 Histological and Immunohistochemical (IHC) Staining

The mice kidney tissues were cut into 3 μm‐thick sections. For histological observations, well‐prepared samples were subjected to periodic Schiff (PAS) and Masson staining. IHC was performed using antibodies diluted as follows: METTL3 (Abcam ab195352, 1:200), MDM2 (Santa Cruz Biotechnology, sc‐965, 1:200), IGF2BP2 (Proteintech, 11601‐1‐AP, 1:200), NICD (Abcam, ab52627, 1:200), Hes1 (Abcam, ab71559, 1:200), and cyclinB1 (Proteintech, 55004‐1‐AP, 1:200). Antibodies were incubated overnight at 4°C, followed by incubation with the appropriate secondary antibodies for 1 h at room temperature. Validation of these antibodies was performed by confirming specific staining in tissues with known expression levels and absence of staining in negative control experiments. After staining with diaminobenzidine and counterstaining with haematoxylin, the sections were visualised under a microscope.

### 2.8 RT‐qPCR

RNA was extracted from kidney tissues or cultured cells using a standard extraction protocol. The extracted RNA was then reverse‐transcribed to cDNA using the Takara TB Green Premix Ex Taq II (Takara, RR820). Real time quantitative PCR (RT‐qPCR) was performed to quantify gene expression levels, using specific primers listed in Table S1.

### 2.9 Flow Cytometry

The apoptotic ratio of cells was assessed using an Annexin V‐FITC/PI Apoptosis Detection Kit according to the manufacturer's protocol (KeyGEN, Nanjing, China). For cell cycle analysis, cells were fixed in 70% cold ethanol, stained with PI staining solution containing RNase A (10 mg/mL), and incubated at 37°C. Cell cycle progression was analysed using an FACScan flow cytometer.

### 2.10 Immunofluorescent (IF) Staining

Frozen kidney tissue sections (3 μm) were fixed with 4% paraformaldehyde for 15 min at room temperature. Podocytes cultured on coverslips were fixed with cold methanol/acetone for 10 min at room temperature. Immunostaining was performed at 4°C with the following primary antibodies and dilutions: Podocin (Invitrogen, PA5‐37904, 1:200); Synap (Sigma‐Aldrich, SAB3500585, 1:200); Ki‐67 (OriGene, TA801577, 1:200); MDM2 (Novus, NBP2‐17247, 1:200); Cyclin B1 (Santa Cruz Biotechnology, sc‐245, 1:200). After incubation with secondary antibodies for 2 h at room temperature, counterstaining of cell nuclei was performed with DAPI (Sigma‐Aldrich) for 10 min. Images were obtained using a confocal microscope.

### 2.11 RNA Immunoprecipitation PCR (RIP) and Sequencing Analysis

RIP assays were performed using the Magna RIP RNA‐Binding Protein Immunoprecipitation Kit (Millipore, 17‐701). Magnetic beads coated with specific antibodies against METTL3 (Proteintech, 10573‐1‐AP), IGF2BP2 (Proteintech, 11601‐1‐AP), or rabbit IgG (Millipore) were incubated overnight with cell lysates. After incubation with proteinase K digestion buffer, RNA‐protein complexes were isolated. PCR was used to analyse the relative interaction between MDM2 and the m6A‐modifying enzymes METTL3 and IGF2BP2.

METTL3‐mediated mRNA from the AGE intervention group and siMETTL3 knockdown group was prepared for next‐generation sequencing. Samples, including those for input controls (IgG) and METTL3 immunoprecipitation, were sequenced by RiboBio (China).

### 2.12 Methylated RNA Immunoprecipitation (MeRIP)‐qPCR

Poly(A) RNA was isolated and fragmented using RNA fragmentation reagents (Thermo, AM8740). For each experiment, one out of every 10 RNA samples was reserved as an input control. Fragmented poly(A) RNA was then incubated with anti‐m6A antibody (Abcam, ab151230) or mouse IgG coupled to Protein A/G Magnetic Beads. This mixture was incubated in 1x poly(A) RNA RNase inhibitor‐supplemented IP buffer and rotated at 4°C for 2 h. The m6A‐containing RNA fragments were subsequently eluted and purified using an RNA purification column. The enrichment of methylated mRNAs was assessed by RT‐qPCR to quantify the levels of specific m6A modifications.

### 2.13 RNA Stability Assays

Actinomycin D (5 μg/mL; MedChemExpress, HY‐17559) was used to halt transcription in cultured podocytes, which were either untreated or treated with siRNA targeting IGF2BP2. Total RNA was extracted at predetermined time intervals (0, 1, 2, 4, and 6 h) following treatment. The stability of MDM2 mRNA was measured by quantifying residual mRNA at each time point using RT‐qPCR.

### 2.14 Luciferase Reporter and Mutation Assay

Mutant versions of MDM2 3′ UTR were generated using a Mut Express II Fast Mutagenesis Kit V2 (Vazyme). Podocytes were transfected with 250 ng of either wild‐type or mutant 3′ UTR luciferase reporter plasmids (Promega). After 48 h, luciferase activity was measured using the Dual Glo Luciferase Assay Kit (Promega), allowing for the assessment of the impact of mutations on gene expression regulation [21].

### 2.15 Statistical Analysis

Quantitative data from independent experiments, performed in triplicate, were expressed as mean ± standard deviation (SD). Statistical significance between two groups was determined using a two‐tailed unpaired Student's *t*‐test, whereas one‐way ANOVA followed by a Bonferroni correction was used for multiple comparisons. All statistical analyses were conducted using SPSS software (version 15.0), with a predetermined significance threshold set at *P <* 0.05.

## 3 Results

### 3.1 METTL3‐Mediated Abnormal m6A Modification in Kidneys of STZ Mice

**FIGURE 1:**
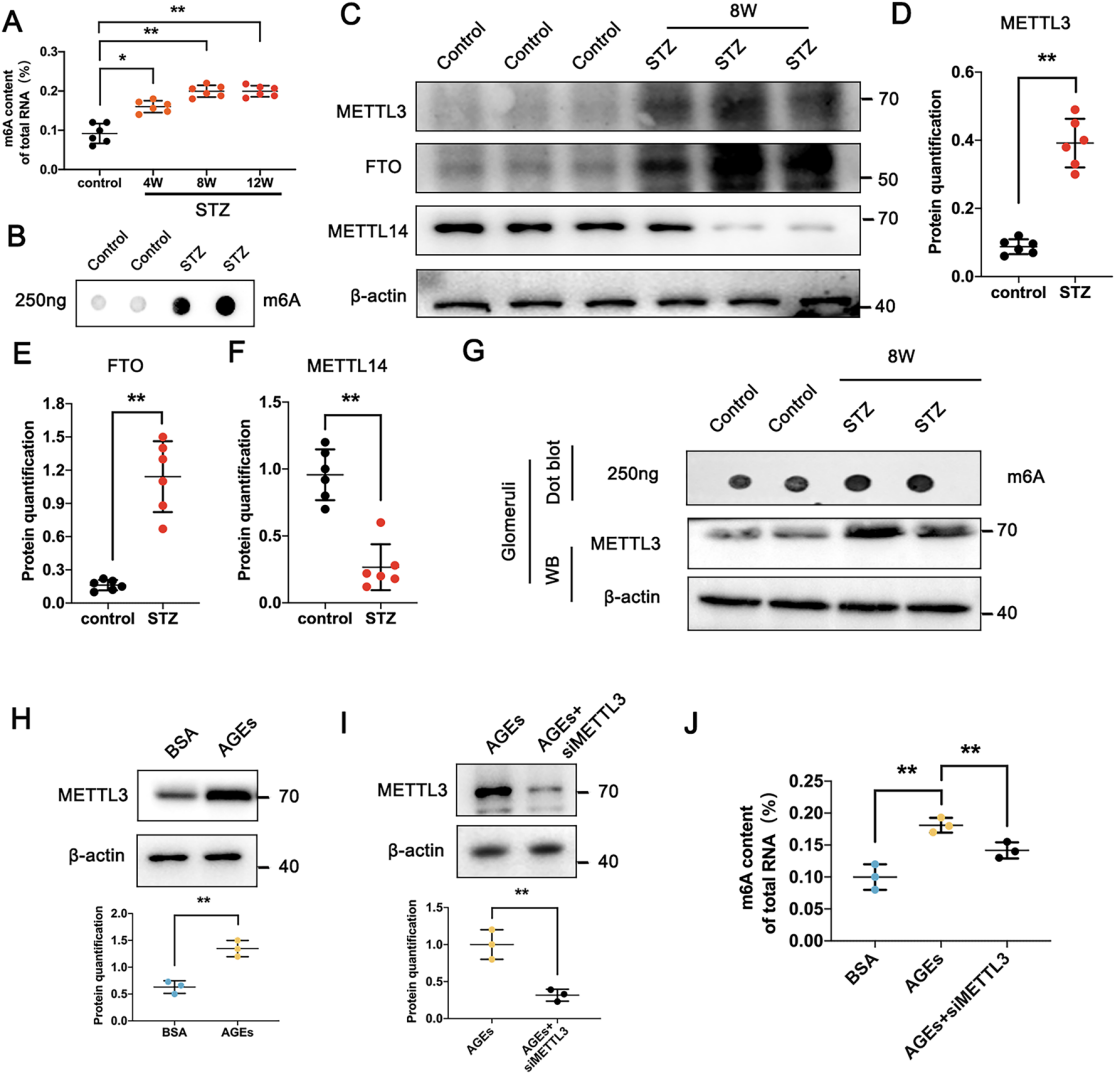
Increased m6A modification and METTL3 protein levels in diabetic mice. (A) Total m6A RNA levels in control and STZ‐treated groups at 4, 8, and 12 weeks (W), as detected by an m6A methylation quantification kit. (B) The m6A level in control and STZ‐treated groups at 12 weeks were detected by dot blot experiment. (C–F) Protein levels of METTL3, FTO and METTL14 in control and STZ‐treated groups analysed by western blot with semi‐quantitative analysis. (G) The m6A level and METTL3 expression of glomeruli in control and STZ‐treated groups. (H) Protein levels of METTL3 in BSA and AGEs‐treated podocytes with semi‐quantitative analysis. (I) Protein levels of METTL3 in AGEs‐treated podocytes with or without siMETTL3 transfection, with semi‐quantitative analysis. (J) Total m6A RNA levels in BSA, AGEs and AGEs‐treated podocytes transfected with siMETTL3, detected by an m6A methylation quantification kit. Data represent mean ± SD of three independent experiments. *p < 0.05 or **p < 0.01 versus control group (A, D–F), or BSA (H), or AGEs (I) by Student's *t*‐test; **p < 0.01 versus AGEs group (J) by one‐way ANOVA.

To assess the role of m6A modification in DKD, we quantified m6A levels in the kidneys of mice at 4, 8, and 12 weeks following treatment with STZ. Both the control and 4‐week STZ groups showed low m6A expression in the kidneys using colorimetric detection. In contrast, m6A levels were significantly higher at 8 and 12 weeks STZ treatment, indicating an increase in expression at these time points (Figure 1A). The result of Dot blot further confirmed the m6A levels of kidney in 12 weeks after STZ induction were significantly higher than those in the control group (Figure 1B). Then, western blot analysis was conducted to define the m6A methyltransferases in kidney during diabetic condition. We observed increased expression of METTL3 and FTO and decreased expression of METTL14 in the STZ‐treated group, suggesting that METTL3 may play a significant role in m6A modification of the kidney under diabetic conditions (Figure 1C–F). Furthermore, the levels of m6A and METTL3 protein expression were both increased in glomeruli of STZ mice (Figure 1G). In podocytes treated with AGEs, METTL3 expression was increased (Figure 1H) and siRNA‐mediated knockdown of METTL3 significantly decreased m6A levels compared to the AGEs group (Figure 1I,J).

### 3.2 Knockdown of METTL3 Attenuated AGEs‐Induced Podocyte Apoptosis and Inflammation

To further understand the biological function of METTL3, podocytes were transfected with siMETTL3 and METTL3 overexpression (OE) plasmid, then co‐cultured with AGEs. Western blot analysis showed that METTL3 knockdown restored the expression of differentiation markers such as synaptopodin (synap) and podocin, increased Bcl2 expression and decreased caspase‐3 cleavage, indicating reduced apoptosis (Figure S1A,B). Additionally, METTL3 inhibition significantly lowered the secretion of pro‐inflammatory cytokines, like TNF‐α, MCP‐1 and IL‐1β in AGEs‐treated podocytes (Figure S1C–E). Flow cytometric analysis further confirmed that siMETTL3 reduced the apoptotic rate in AGEs‐treated podocytes (Figure S1F). Conversely, METTL3 overexpression exacerbated the release of TNF‐α, MCP‐1 and IL‐1β and increased apoptosis under AGEs conditions (Figure S1G,H). These findings suggest that METTL3 plays a crucial role in mediating podocyte injury under diabetic conditions.

### 3.3 Conditional METTL3 Knockout Protected Against Renal Damage in STZ‐Induced Diabetic Mice

**FIGURE 2:**
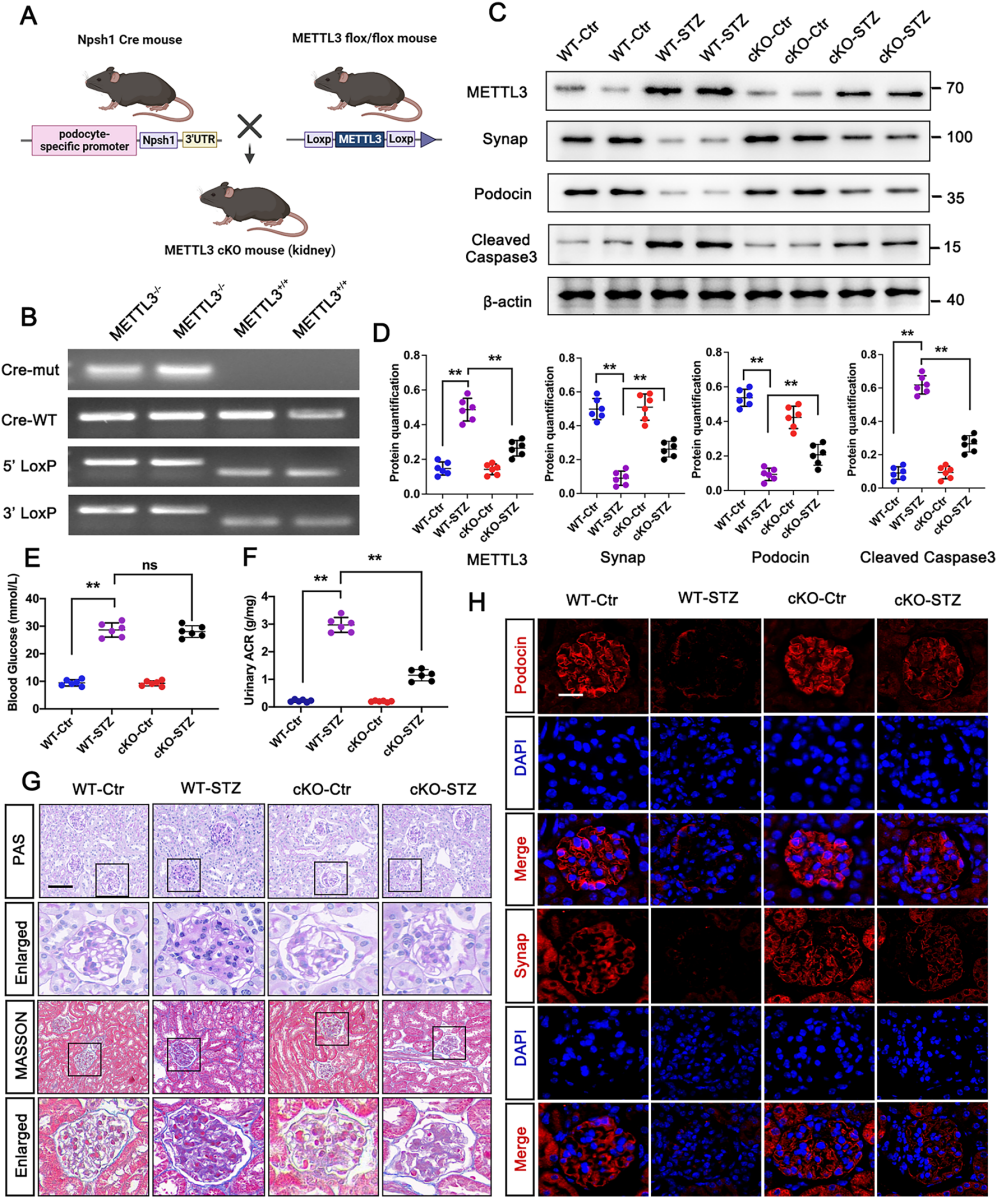
Conditional knockout of podocytes METTL3 significantly alleviates kidney damage in diabetic mice. (A) Schematic representation of the genetic approach used to develop a conditional podocyte‐specific METTL3 knockout mouse model. (B) Genotype identification by tail PCR. (C, D) Protein levels of Synaptopodin (Synap), Podocin, and cleaved caspase 3 in WT control (WT‐Ctr), WT STZ‐treated (WT‐STZ), conditional knockout control (cKO‐Ctr) and conditional knockout STZ‐treated (cKO‐STZ) groups, analysed by western blot with semi‐quantitative analysis. (E, F) Blood glucose levels and urinary albumin/creatinine ratio (UACR) in WT‐Ctr, WT‐STZ, cKO‐Ctr and cKO‐STZ groups. (G) Masson and PAS staining in WT‐Ctr, WT‐STZ, cKO‐Ctr and cKO‐STZ groups; scale bars = 50 μm. (H) IF staining of Podocin (red) and Synap (red), counterstained with DAPI (blue) in WT‐Ctr, WT‐STZ, cKO‐Ctr and cKO‐STZ groups; scale bars = 20 μm. Data represent mean ± SD of three independent experiments. **p < 0.01 versus WT‐STZ group by one‐way ANOVA.

To investigate the in vivo function of METTL3, we developed a conditional podocyte‐specific METTL3 knockout (cKO) mouse model. Utilising the Cre‐LoxP recombination system, Nphs1‐Cre METTL3^fl/fl^ mice were generated by breeding Nphs1‐Cre mice with METTL3^fl/fl^ mice, resulting in targeted deletion of METTL3 in podocytes (Figure 2A). This deletion was confirmed via tail genotyping and western blot analysis of METTL3 expression (Figure 2B–D). Western blot analysis and immunofluorescence staining demonstrated a marked increase in the expression of podocyte differentiation markers, including podocin and synaptopodin in the METTL3 knockout group compared to the wild‐type STZ‐treated (WT‐STZ) group (Figure 2C,D). Additionally, PAS and Masson's trichrome staining revealed that METTL3 deficiency significantly reduced STZ‐induced glomerular sclerosis and extracellular matrix accumulation, alongside a decrease in the albumin/creatinine ratio (UACR) (Figure 2F,G). Interestingly, METTL3 deletion in podocytes did not affect blood glucose levels relative to the WT‐STZ group (Figure 2E). Furthermore, the specific deletion of METTL3 in podocytes showed no significant alterations in renal pathology, UACR and podocyte differentiation markers when compared to the wild‐type (WT) group, underscoring the specific protective effects of METTL3 deletion against podocyte injury in the context of DKD (Figure 2C–H). These findings collectively demonstrate the potential therapeutic benefits of targeting METTL3 in protecting podocytes under diabetic conditions.

### 3.4 Abnormal m6A Modification of MDM2 Is Regulated by METTL3

**FIGURE 3:**
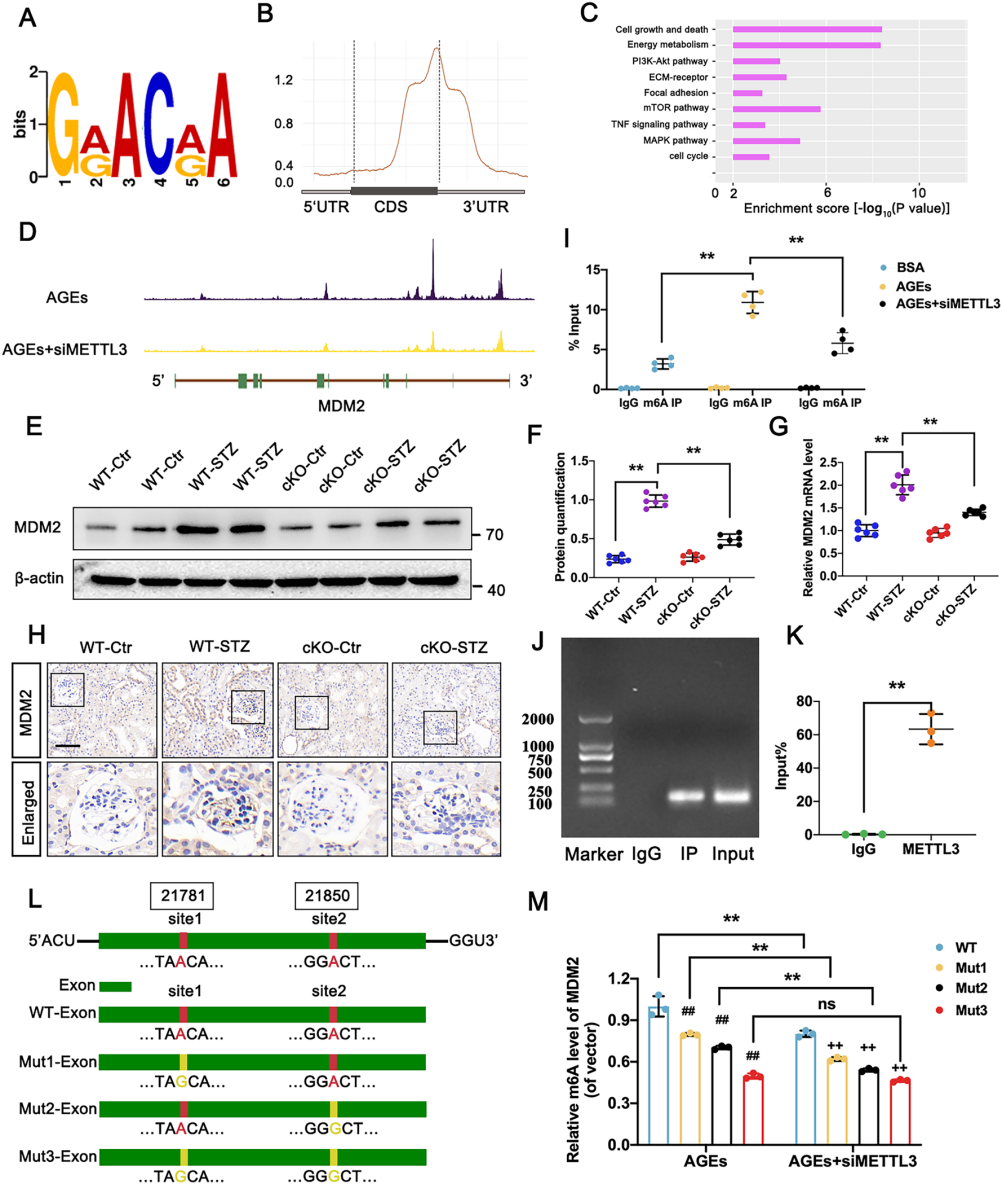
Identification of METTL3 targets in podocytes. (A) Motif analysis of METTL3‐binding sites from RIP‐seq in AGEs‐treated podocytes and AGEs‐treated podocytes transfected with siMETTL3. (B) Peak distribution of gene regions in METTL3‐modified transcripts. (C) GO analysis of genes modified by METTL3. (D) M6A abundances in MDM2 transcripts in AGEs‐treated podocytes and AGEs‐treated podocytes transfected with siMETTL3. (E) Enriched m6A modification of MDM2 in BSA‐treated, AGEs‐treated and AGEs‐treated podocytes transfected with siMETTL3, determined by MeRIP‐qPCR assay. (F, G) Protein levels of MDM2 in WT‐Ctr, WT‐STZ, cKO‐Ctr, and cKO‐STZ groups, analysed by western blot with semi‐quantitative analysis. (H) MDM2 mRNA levels in WT‐Ctr, WT‐STZ, cKO‐Ctr and cKO‐STZ groups, determined by RT‐qPCR. (I) Protein levels of MDM2 in WT‐Ctr, WT‐STZ, cKO‐Ctr and cKO‐STZ groups, analysed by IHC assay; scale bar = 50 μm. (J, K) Direct interaction between MDM2 and METTL3 in podocytes, demonstrated by RIP‐PCR assay. (L) Mutations at the two putative m6A sites in MDM2 (A–G). (M) m6A levels of MDM2 in podocytes treated with AGEs, co‐expressing siMETTL3 and MDM2‐WT/Mutants, determined by MeRIP‐qPCR. Data represent mean ± SD of three independent experiments. **p < 0.01 versus AGEs group (E), or WT‐STZ group (G) by one‐way ANOVA; **p < 0.01 versus IgG group (K) by Student's *t*‐test; ##*p* < 0.01 versus AGEs‐WT group; ++*p* < 0.01 versus AGEs+siMETTL3‐WT group; **p < 0.01 versus AGEs group by two‐way ANOVA.

To elucidate the molecular mechanisms underlying METTL3‐induced podocyte injury in DKD, we performed RNA‐IP sequencing (RIP‐seq) on podocytes treated with AGEs. RIP‐seq data indicated that m6A modifications were predominantly found in the consensus “RRACH” motif (*R* = G or A, H = A, C, or U). These modifications were notably enriched near the 3′ untranslated regions and termination codons of coding DNA sequences, aligning with prior findings [22] (Figure 3A,B). Kyoto Encyclopedia of Genes and Genomes pathway analysis revealed that genes highly methylated by METTL3 were mainly involved in pathways related to cell cycle regulation and cellular survival (Figure 3C). Notably, MDM2, a key player in cell survival pathways, showed significant METTL3‐mediated m6A modifications. A visual decrease in m6A modification peaks was observed in the 3′ UTR of MDM2 in METTL3‐deficient samples (Figure 3D). Western blot, IHC and RT‐qPCR analysis demonstrated that silencing METTL3 led to a reduction in MDM2 expression compared to the WT‐STZ group (Figure 3E–H). Furthermore, MeRIP‐qPCR analysis showed that m6A levels in MDM2 mRNA were substantially lower in podocytes transfected with siMETTL3 compared to those treated with AGEs (Figure 3I). RIP‐PCR assays confirmed a direct interaction between MDM2 mRNA and METTL3, validating the specificity of m6A modification by METTL3 (Figure 3J,K).

Analysis of m6A sites in MDM2 using the SRAMP prediction tool highlighted two potential modification sites. Mutation analysis, converting adenine to guanine at these sites (MDM2‐Mut1 and MDM2‐Mut2), was performed to study their impact on METTL3 binding. Results from MeRIP‐qPCR indicated that METTL3 downregulation decreased m6A modifications in MDM2‐WT, MDM2‐Mut1 and MDM2‐Mut2 constructs in podocytes under AGEs conditions. However, the MDM2‐Mut3 construct, containing mutations at both m6A sites, did not show significant changes in m6A levels after METTL3 silencing (Figure 3M). This suggests that mutations at these sites impact the binding efficiency between MDM2 mRNA and METTL3, indicating METTL3‐mediated MDM2 m6A demethylation have key roles in DKD.

### 3.5 IGF2BP2 Promotes MDM2 mRNA Stability in an m6A‐Dependent Manner

**FIGURE 4:**
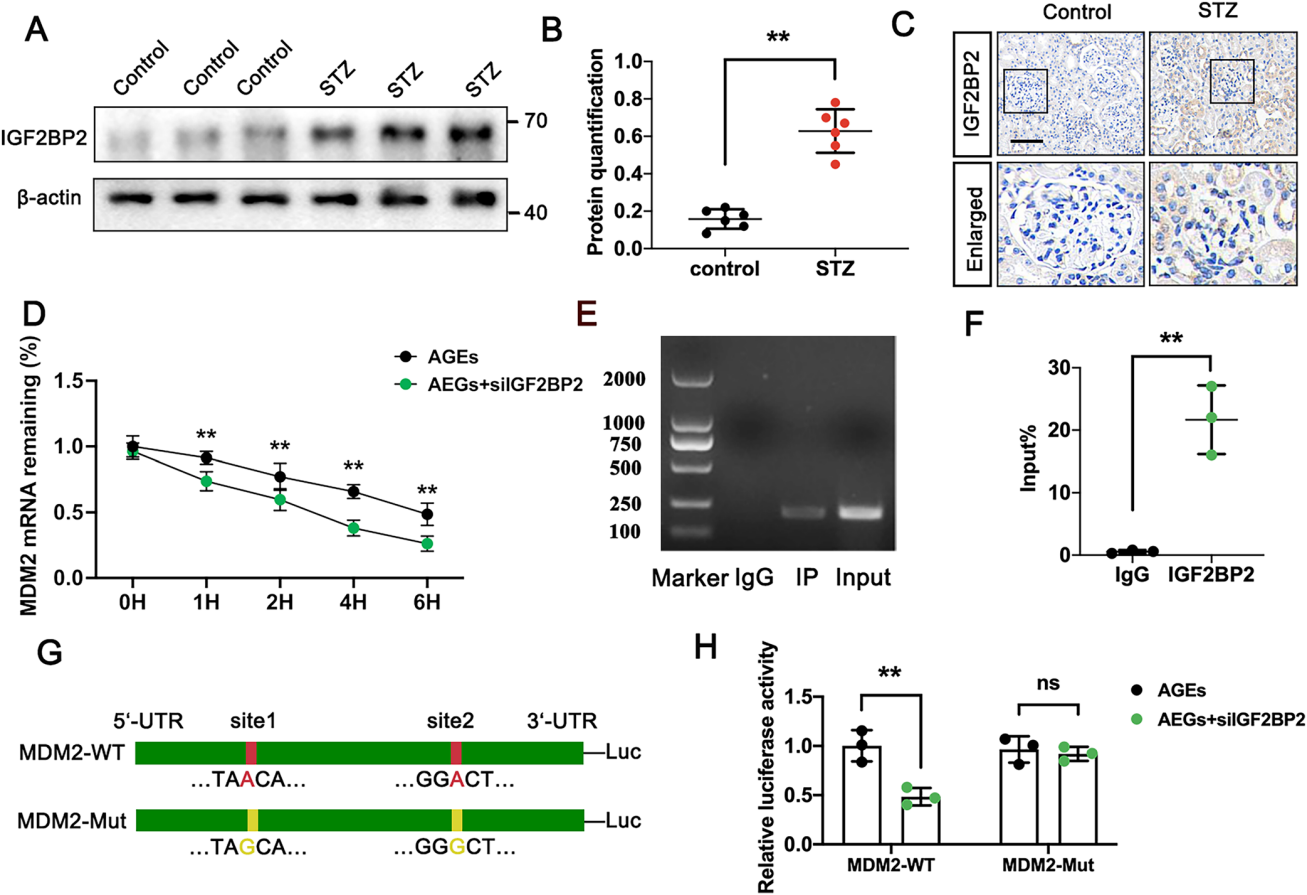
IGF2BP2 identifies m6A‐meditated MDM2 mRNA and participates in MDM2 mRNA stability. (A, B) Protein levels of IGF2BP2 in control and STZ‐treated groups, analysed by western blot with semi‐quantitative analysis. (C) Protein levels of IGF2BP2 in control and STZ‐treated groups, determined by IHC assay; scale bar = 50 μm. (D) MDM2 mRNA levels in podocytes treated with actinomycin D in AGEs‐treated and AGEs‐treated podocytes transfected with siIGF2BP2, determined by RT‐qPCR. (E, F) Direct interaction between MDM2 and IGF2BP2 in podocytes, demonstrated by RIP‐PCR assay. (G, H) Relative luciferase activity of the MDM2‐WT or MDM2‐Mut 3′UTR luciferase reporter in AGEs‐treated and AGEs‐treated podocytes transfected with siIGF2BP2. Data represent mean ± SD of three independent experiments. **p < 0.01 versus STZ group (B), AGEs group (H), or IgG group (F) by Student's t‐test.

Recent studies have indicated that m6A modifications are recognised by specific “reader” proteins, which subsequently affect downstream biological functions [23]. Notably, IGF2BP2 has been reported to enhance mRNA stability by recognising m6A motifs in the context of DKD [24]. In this study, we investigated whether IGF2BP2 is involved in recognising METTL3‐mediated m6A modifications on MDM2 mRNA in podocytes. Western blot analysis revealed significant upregulation of IGF2BP2 in the kidney tissues of the STZ‐treated group (Figure 4A,B). IHC staining further confirmed elevated IGF2BP2 levels in these samples (Figure 4C). To assess the functional effect of IGF2BP2 in mRNA stability, we performed RNA stability assays of MDM2 mRNA using actinomycin D to halt transcription in AGEs‐treated podocytes. Results indicated that depletion of IGF2BP2 markedly decreased the stability of MDM2 mRNA (Figure 4D), suggesting that IGF2BP2 plays a critical role in stabilising MDM2 transcripts. Furthermore, RIP assays demonstrated a specific interaction between MDM2 mRNA and IGF2BP2, confirming the binding of IGF2BP2 to m6A‐modified sites on MDM2 mRNA (Figure 4E,F). Additionally, a luciferase reporter assay was employed to directly observe the binding dynamics of IGF2BP2 to the m6A sites on MDM2 mRNA (Figure 4G,H). Our findings exhibit that IGF2BP2 directly interacts with m6A‐modified MDM2 mRNA, enhancing its stability in an m6A‐dependent manner.

### 3.6 MDM2 Contributes to Podocyte Injury Under Diabetic Condition

Murine Double Minute 2 (MDM2), a crucial regulator of the cell cycle pathway, has been implicated in high glucose‐induced podocyte cell cycle arrest and dedifferentiation [25]. In our study, we explored the effect of MDM2 on podocytes under high glucose conditions. AGEs exposure led to cell cycle arrest of podocytes, characterised by increased expression of PCNA, P21 and Cyclin B1 for S, G2/M and M phases respectively (Figure S2A,B). Flow cytometry analysis further demonstrated an increased percentage of podocytes transitioning from the G0/G1 phase to S and G2/M phases following AGEs treatment (Figure S2C). Additionally, siMDM2 effectively inhibited the re‐entry of podocytes into the AGEs‐induced G2/M phase, maintaining them in the G0/G1 phase (Figure S2A–C). Moreover, as demonstrated in Figure S2A,B, western blot analysis confirmed that damaged podocytes in the AGEs group were repaired by upregulating the levels of Synap levels with MDM2 inhibition.

**FIGURE 5:**
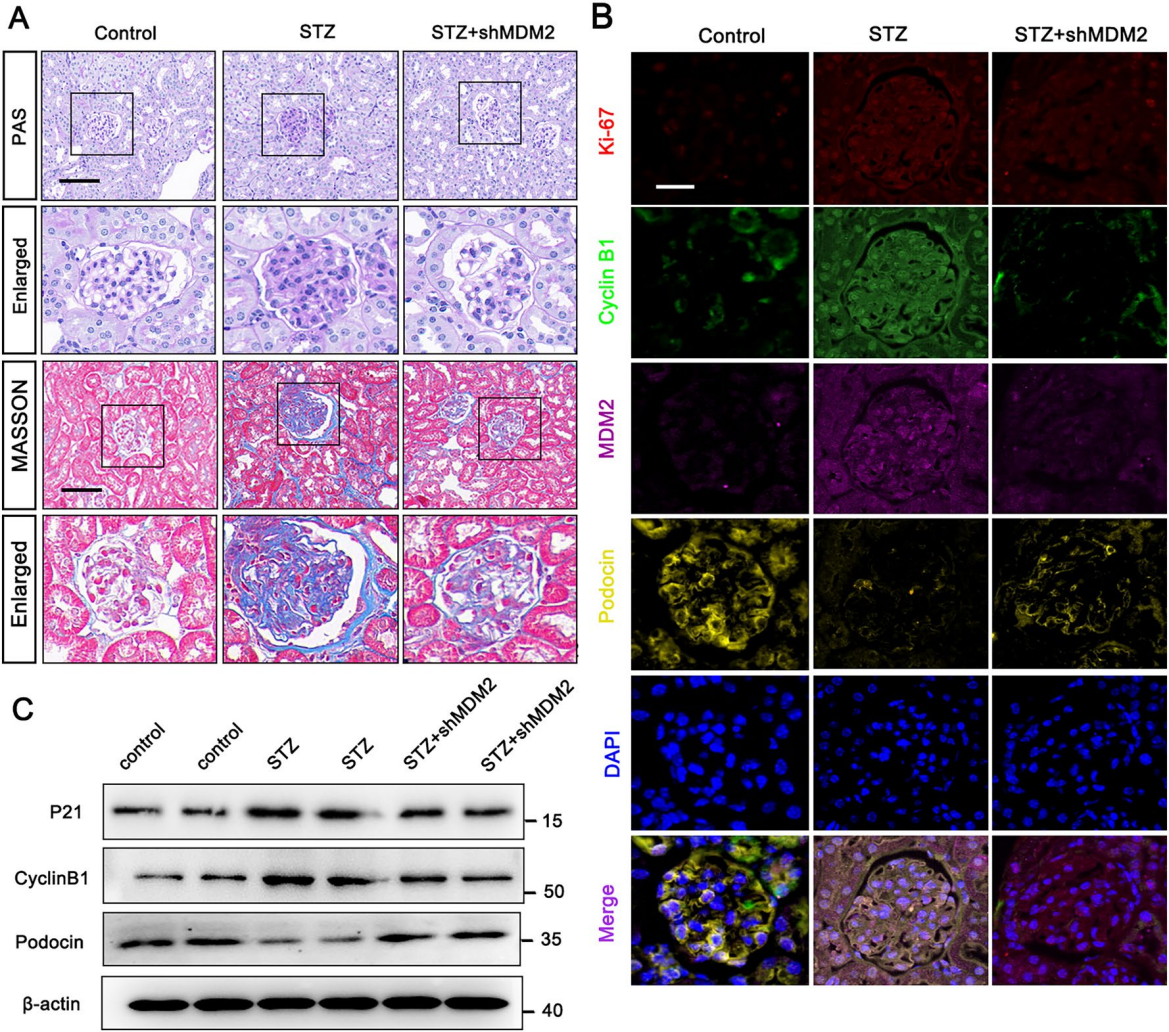
Inhibiting podocytes MDM2 expression alleviates renal pathological damage, and reduces podocytes dedifferentiation levels. (A) Representative images showing PAS and Masson staining (scale bar = 20 μm) in control, STZ, and shMDM2‐injected STZ groups. (B) IF staining of Ki‐67 (red), Cyclin B1 (green), Podocin (yellow), and MDM2 (pink), counterstained with DAPI (blue) in control, STZ and STZ injected with shMDM2 groups. (C) Protein levels of p21, Cyclin B1 and Podocin in control, STZ and STZ injected with shMDM2 groups, analysed by western blot. Data represent mean ± SD of three independent experiments.

To examine the effects of MDM2 in vivo, we utilised AAV9‐shMDM2 to silence MDM2 in the kidneys of diabetic mice. Histological analysis via PAS and Masson's trichrome staining revealed that MDM2 silencing significantly reduced renal fibrosis (Figure 5A). Additionally, fluorescence intensity of podocin increased while Ki‐67, Cyclin B1, and MDM2 decreased in the shMDM2 group compared to the STZ group (Figure 5B). Western blot results confirmed the reduced expression of p21 and Cyclin B1, and increased Podocin levels in shMDM2 group compared to the STZ group (Figure 5C). These findings collectively suggest that MDM2 plays a critical role in podocyte injury caused by the diabetic condition.

### 3.7 MDM2 Inhibition Mitigates Notch1 Signalling Pathway of Podocytes Under Diabetic Condition

**FIGURE 6:**
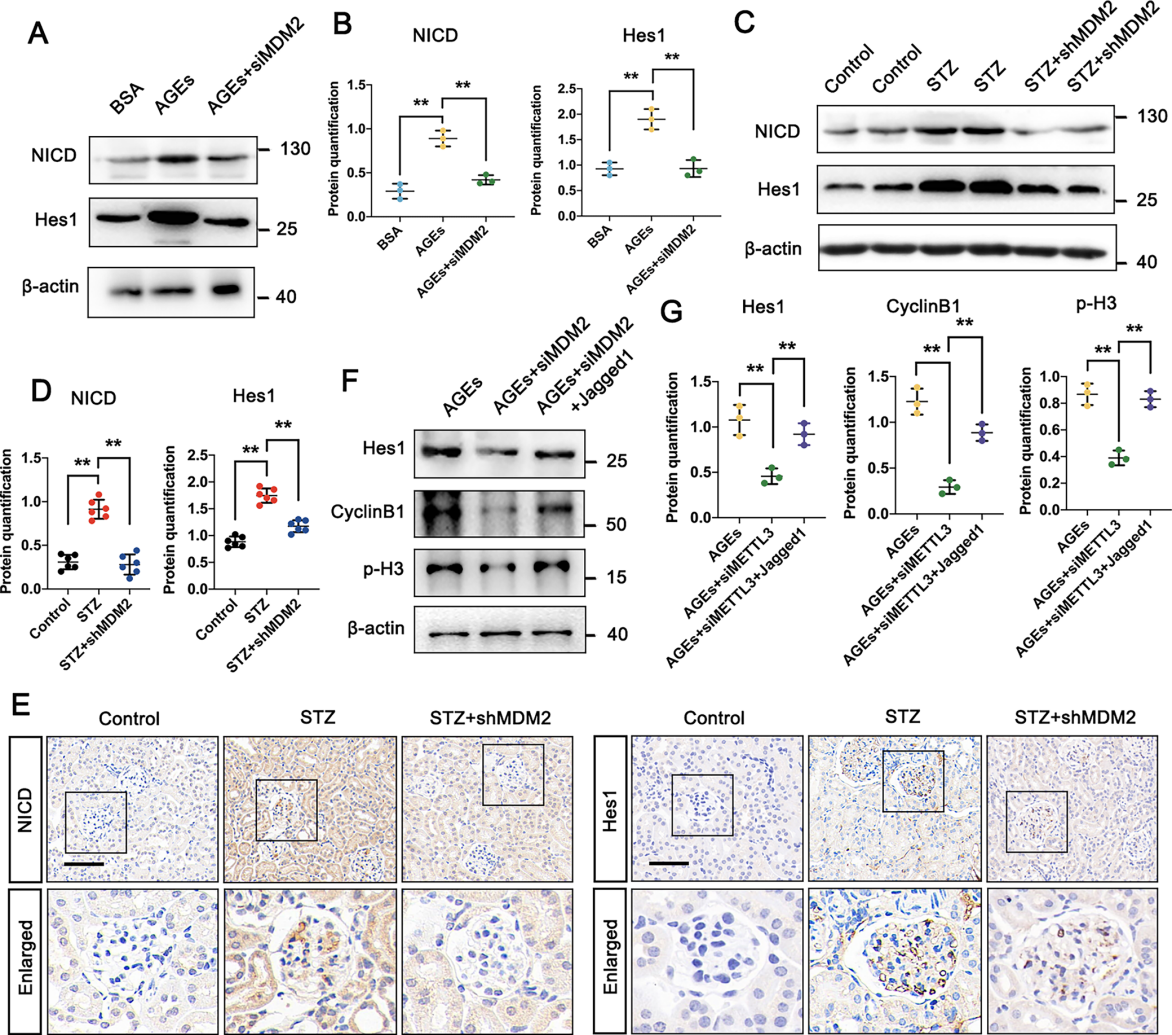
MDM2 regulates the Notch1 signalling pathway in podocytes during AGE‐induced abnormal cell cycle regulation. (A, B) Western blot analysis and semi‐quantitative assessment of NICD and Hes1 protein levels in BSA‐treated, AGE‐treated, and siMDM2‐transfected AGE‐treated groups. (C, D) Western blot analysis and semi‐quantitative assessment of NICD and Hes1 protein levels in control, STZ‐treated, and shMDM2‐injected STZ‐treated groups. (E) Evaluation of NICD and Hes1 protein levels in control, STZ‐treated and shMDM2‐injected STZ‐treated groups by IHC assay (scale bar = 50 μm). (F, G) Western blot analysis and semi‐quantitative assessment of Hes1, Cyclin B1 and p‐H3 protein levels in AGE‐treated, AGE + siMDM2‐treated, and Jagged1‐treated AGE + siMDM2 groups. Data represent mean ± SD of three independent experiments. **p < 0.01 versus AGE group (B), or STZ group (D), or AGE + siMDM2 group (G) by one‐way ANOVA.

MDM2 is known to play a critical role in cell differentiation by regulating the downstream Notch1 signalling pathway during organismal development. In our study, silencing MDM2 effectively suppressed the upregulation of NICD and subsequent activation of the target gene Hes1, which were induced by AGEs (Figure 6A,B). Western blot analysis and IHC staining in STZ‐treated mice injected with AAV‐NPHS‐shMDM2 revealed that MDM2 knockout decreased the expression of NICD and Hes1 in kidney tissues, suggesting a reduction in Notch1 pathway activity (Figure 6C–E). To further validate the role of the Notch1 signalling pathway as downstream of MDM2‐mediated podocyte cell cycle arrest, podocytes were co‐cultured with AGEs and Jagged1 (a known Notch pathway activator) or transfected with siMDM2. Inhibition of Notch signalling resulted in decreased levels of Hes1 and inhibition of cell cycle arrest compared to the AGEs group (Figure 6F,G). These results indicate that MDM2 regulated the Notch1 signalling pathway, contributing to abnormal cell cycle dynamics in podocytes under diabetic conditions.

### 3.8 METTL3 Alleviated Podocyte Injury Through Notch Signalling Pathway

Further investigations were conducted to elucidate the effect of METTL3 on the MDM2/Notch pathway and podocyte injury. Flow cytometry analysis further demonstrated the decreased accumulation arrest in S and G2/M in podocytes with siMETTL3 compared to the AGEs group (Figure S3A). Additionally, IF staining revealed reduced NICD fluorescence intensity in podocytes transfected with siMETTL3 compared to the AGE‐treated group (Figure S3C). IHC staining further demonstrated that specific deletion of METTL3 in podocytes decreased the expression of Notch1 signalling proteins such as NICD and Hes1, along with reduced expression of Cyclin B1 in the glomeruli compared to the STZ group (Figure S3B). These findings indicate that METTL3 regulates podocyte injury through the m6A modification of MDM2, which subsequently affects the downstream Notch signalling pathway.

## 4 Discussion

Previous studies have extensively explored the role of METTL3 in various cell types under DKD conditions, including renal tubular epithelial cells [26, 27]. However, research on METTL3 in glomerular podocytes remains limited. We hypothesise that METTL3 may also play an important role in glomerular podocytes during the progression of DKD. This study expands upon previous findings by incorporating a glomerular podocyte‐specific analysis of METTL3, which could help uncover new therapeutic targets for DKD.

In this study, we found that the expression of METTL3 was significantly increased in both podocytes under high glucose conditions in diabetic mice induced by STZ. The upregulation of METTL3 contributes to podocyte injury and the release of inflammatory factors under diabetic conditions, aggravating renal pathology and increasing urinary albumin excretion. Mechanistically, increased METTL3 enhanced the m6A RNA methylation of MDM2 and increased its RNA stability through the IGF2BP2‐dependent pathway. Knockdown of MDM2 alleviates podocyte injury driven by Notch signalling. These findings collectively suggest that targeting the METTL3/MDM2 axis may be an attractive target for DKD.

M6A modification is a critical post‐transcriptional modification in eukaryotic mRNA and plays a significant role in the pathogenesis of various diseases [28, 29]. However, research on m6A methylation in DKD has not been fully elucidated. Our study observed the elevated expression of METTL3 and FTO in STZ‐treated mice. Considering the rising m6A RNA levels and the most evident upregulation of METTL3 expression in kidneys, methyltransferase METTL3 was chosen as the target in our study. Further investigations would be needed to find out whether METTL3 contributes to the progression of podocytopathies. Conditional knockout of METTL3 in podocytes notably reduced apoptosis and inflammatory cytokine release under diabetic conditions and alleviated podocyte dedifferentiation, renal pathological damage, and urinary albumin excretion. In contrast, overexpression of METTL3 in podocytes exacerbated apoptosis and increased the release of inflammatory cytokines. Our study findings are consistent with the previous studies that reveal the pathogenic effect of METTL3 in podocytopathies [24, 30].

To further identify downstream genes modified by METTL3 that contribute to podocyte injury, we performed RIP‐seq, RIP‐PCR and MeRIP‐qPCR analyses to confirm the effect of METTL3 on MDM2 expression through m6A modification. MDM2, an E3 ubiquitin ligase, plays a crucial role in cell cycle regulation, tumorigenesis and cellular differentiation through both p53‐dependent and p53‐independent pathways [31, 32]. The role of MDM2 in kidney diseases has been studied extensively, and we described the pathogenic effect of MDM2 in DKD here, showing that inhibition of MDM2 alleviated pathological injury in podocytes under diabetic conditions. However, published studies show that the pathologic role of MDM2 in kidney disease is controversial and that its inhibition may not always be beneficial [33]. Cell death and progression of glomerular injury induced by podocyte‐specific MDM2 ablation in mice can be suppressed in double‐transgenic animals by dual p53 and MDM2 silencing in podocytes [34]. A recent study demonstrated that MDM2 expression is dramatically attenuated in patients with diabetic nephropathy and in vitro or in vivo experiments [35, 36, 37]. The possible reason is that the effect of MDM2 is diverse in different renal parenchymal cells.

The Notch1 pathway is responsible for cell differentiation during organism development [38]. Its aberrant activation in mature kidneys is considered an important mechanism in the progression of proteinuria caused by glomerular injury and podocyte lesions [39]. Previous studies demonstrated that MDM2 activated the Notch signalling pathway by non‐degradative ubiquitination and synergised with Notch1 to inhibit apoptosis and promote proliferation [40]. Additionally, differentiated podocytes face inherent obstacles to mitosis, which weakens their proliferative response and impedes recovery from injury [41, 42]. Recent studies suggest that dis‐regulation in podocyte cell cycle regulation and subsequent damage may occur in DKD, particularly in response to chronic hyperglycaemia and metabolic stress [43, 44]. Consistent with these findings, our data showed that podocytes undergo cell cycle arrest under diabetic conditions. These abnormal cell cycle events were driven by the activation of METTL3‐mediated MDM2‐Notch1 signalling under diabetic conditions, enabling mature podocytes to overcome the G2/M checkpoint. METTL3 knockout inhibited the abnormal activation of the MDM2‐Notch1 pathway, preventing podocyte death during the initial phases of glomerular injury and alleviating glomerulosclerosis.

We further investigated the underlying mechanisms through which METTL3 mediates podocyte injury. The stability and function of target transcripts are influenced by m6A readers, including IGF2BP1, IGF2BP2 and IGF2BP3. These m6A readers specifically recognise m6A‐modified mRNA transcripts by identifying a conserved GGC (m6A) sequence, thereby influencing mRNA stability, protein expression, and exerting regulatory roles under stress conditions [45, 46]. Unlike the YTH domain‐containing family protein 2, which promotes mRNA degradation, IGF2BP2 enhances mRNA stability [46, 47]. A previous study found IGF2BP2 could recognise METTL3‐mediated m6A modifications on MIS12, enhancing the stability and expression of MIS12, reversing senescence in human mesenchymal stem cells [48]. In our study, increased expression of IGF2BP2 was observed in the renal tissues of DKD mice. IGF2BP2 directly associates with specific m6A sites on MDM2 mRNA, maintaining MDM2 mRNA stability under the regulation of METTL3.

Recent studies on acute injury have explored the therapeutic potential of Cpd‐564, a novel METTL3 inhibitor, which showed remarkable efficacy in restoring kidney function, inhibiting kidney injury, and alleviating inflammatory responses [22, 49]. Previous studies have shown that METTL3 plays a significant role in the development of polycystic kidney and chronic kidney disease, but there are few reports on its role in DKD [16, 50]. Recent studies have found that increased METTL3 expression contributed to the upregulated m6A modification levels in DKD. Moreover, knockout of METTL3 has been shown to alleviate kidney injury [24]. Compared to previous studies, we used a podocyte‐specific METTL3 gene knockout mouse model, which highlights the pivotal role of m6A modification in podocyte injury. Moreover, by investigating the relationship between METTL3‐mediated m6A modification and its target gene MDM2, as well as the Notch signalling pathway, this study provides further insight into the specific mechanisms of m6A modification in the progression of DKD. Our study provides a novel theoretical foundation for m6A modification as a new therapeutic target for DKD. Future research should continue to explore the therapeutic potential of METTL3 inhibitors and their impact on podocyte function and renal pathology. Additionally, we did not conduct further detection of METTL3 in clinical samples here, and it is necessary to further study whether m6A modification can be used as a diagnostic label for DKD.

## 5 Conclusions

In conclusion, our study found elevated expression of METTL3 in both podocytes under high glucose conditions and the kidneys of diabetic mice models. Increased METTL3 induced abnormal m6A modification of MDM2 through an IGF2BP2‐dependent mechanism. The enhanced m6A modification subsequently triggers inflammation and apoptosis in podocytes via the upregulation of the Notch1 signalling pathway. Our findings indicate dysregulated RNA m6A modification mediated by METTL3 may be a promising target for the diagnosis and therapy of DKD.

## Author Contributions

**Han Wu:** conceptualization (equal). **Ziyang Yu:** writing – original draft (equal). **Yitian Yang:** writing – original draft (equal). **Zhuoting Han:** data curation (equal). **Qingjun Pan:** data curation (equal), formal analysis (equal). **Ying Chen:** writing – review and editing (equal). **Hongyuan Yu:** writing – review and editing (equal). **Siman Shen:** conceptualization (equal), writing – review and editing (equal). **Li Xu:** project administration (lead).

## Ethics Statement

All studies were approved by the Ethics Committee on the Care and Use of Laboratory Animals of China Medical University (KT2020148).

## Consent

All authors have read and approved of their submission to this journal.

## Conflicts of Interest

The authors declare no conflicts of interest.

## Supporting information


Data S1

## Data Availability

Data that are not already included in the manuscript is available upon request.
