# Supplementary material for: The Impact of METTL3 on MDM2 Promotes Podocytes Injury During Diabetic Kidney Disease
Source: J Cell Mol Med. 2025 May 27;29(10):e70627. doi: 10.1111/jcmm.70627 (PMC12107706; doi:10.1111/jcmm.70627)
Supplement: Supplementary file 1 — Data S1 [file JCMM-29-e70627-s001.docx]

**Supplemental Table S1**

Table S1. Primers for RT-qPCR.

| **Primer name** |  | **Sequence** |
| --- | --- | --- |
| mmu-TNFα | Sense | ACGCTCTTCTGTCTACTGAACTTCG |
|  | Anti-sense | TGGTTTGTGAGTGTGAGGGTCTG |
| mmu-MCP-1 | Sense | ACTCACCTGCTGCTACTCATTCAC |
|  | Anti-sense | TCTTTGGGACACCTGCTGCTG |
| mmu-IL-1β | Sense | CTCGCAGCAGCACATCAACAAG |
|  | Anti-sense | CCACGGGAAAGACACAGGTAGC |
| mmu-MDM2 | Sense | TCAGGCAGAAGAAGGCTTGGATG |
|  | Anti-sense | ATGGTTGGGAATAGTCGTCACTCTC |
| mmu-β-actin | Sense | AGAGGGAAATCGTGCGTGAC |
|  | Anti-sense | CAATAGTGATGACCTGGCCGT |

**Supplemental Figures**

**
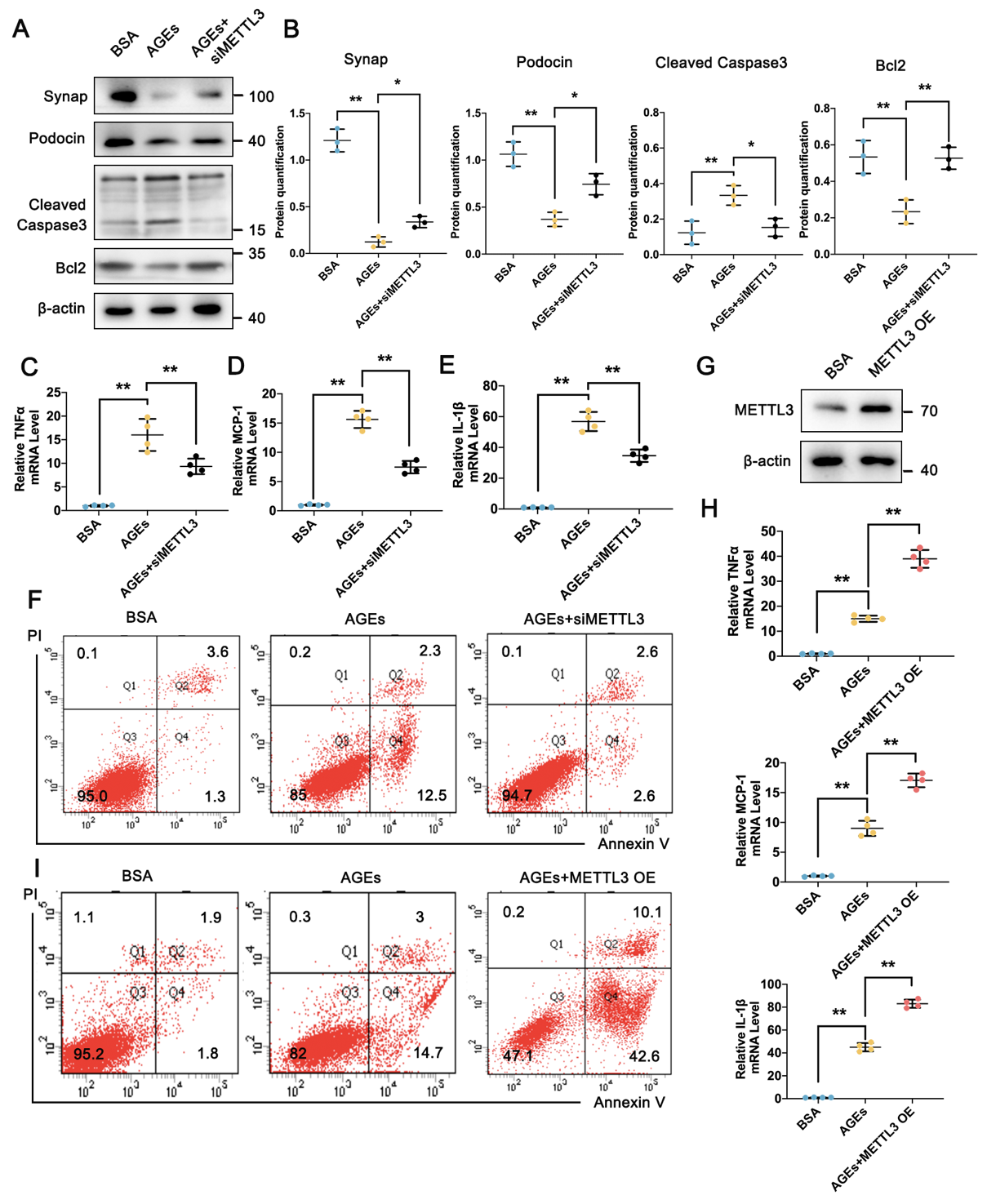
**

**Figure S1. METTL3 is involved in AGE-induced apoptosis of podocytes and release of inflammatory factors**

(A-B) Western blot analysis and semi-quantitative assessment of synaptopodin (Synap), podocin, caspase 3, and Bcl2 protein levels in BSA-treated, AGE-treated, and AGE + siMETTL3-transfected groups. (C-E) mRNA levels of TNF-α, MCP-1, and IL-1β in BSA-treated, AGE-treated, and AGE + siMETTL3-transfected groups. (F) Podocytes stained with FITC-Annexin V and PI, analyzed using flow cytometry in BSA-treated, AGE-treated, and AGE + siMETTL3-treated groups. (G) Western blot analysis of METTL3 protein levels in vector and METTL3 OE groups. (H) mRNA levels of TNF-α, MCP-1, and IL-1β in BSA-treated, AGE-treated, and METTL3 OE groups. (I) Podocytes stained with FITC-Annexin V and PI, analyzed using flow cytometry in BSA-treated, AGE-treated, and METTL3 OE groups. Data represent mean ± SD of three independent experiments. *P<.05 or **P<.01 versus AGE group by one-way ANOVA.


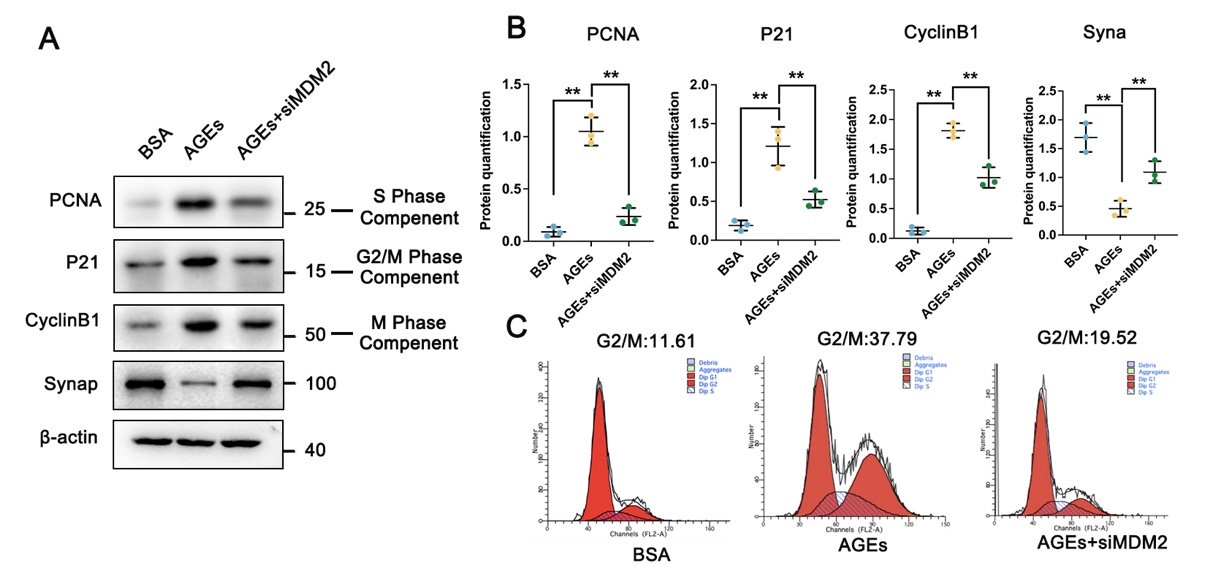


**Figure S2. MDM2 knock-out inhibits AGE-induced mitotic catastrophe in podocytes**

(A-B) Analysis of protein levels of PCNA, P21, Cyclin B1 and Synap in BSA-treated, AGE-treated, and AGE-treated with siMETTL3 groups, performed using western blotting followed by semi-quantitative assessments. (C) Assessment of cell cycle events in podocytes from BSA-treated, AGE-treated, and AGE-treated with siMETTL3 groups using flow cytometry. Data represent mean ± SD of three independent experiments. P<.01 versus AGE group analyzed by one-way ANOVA.


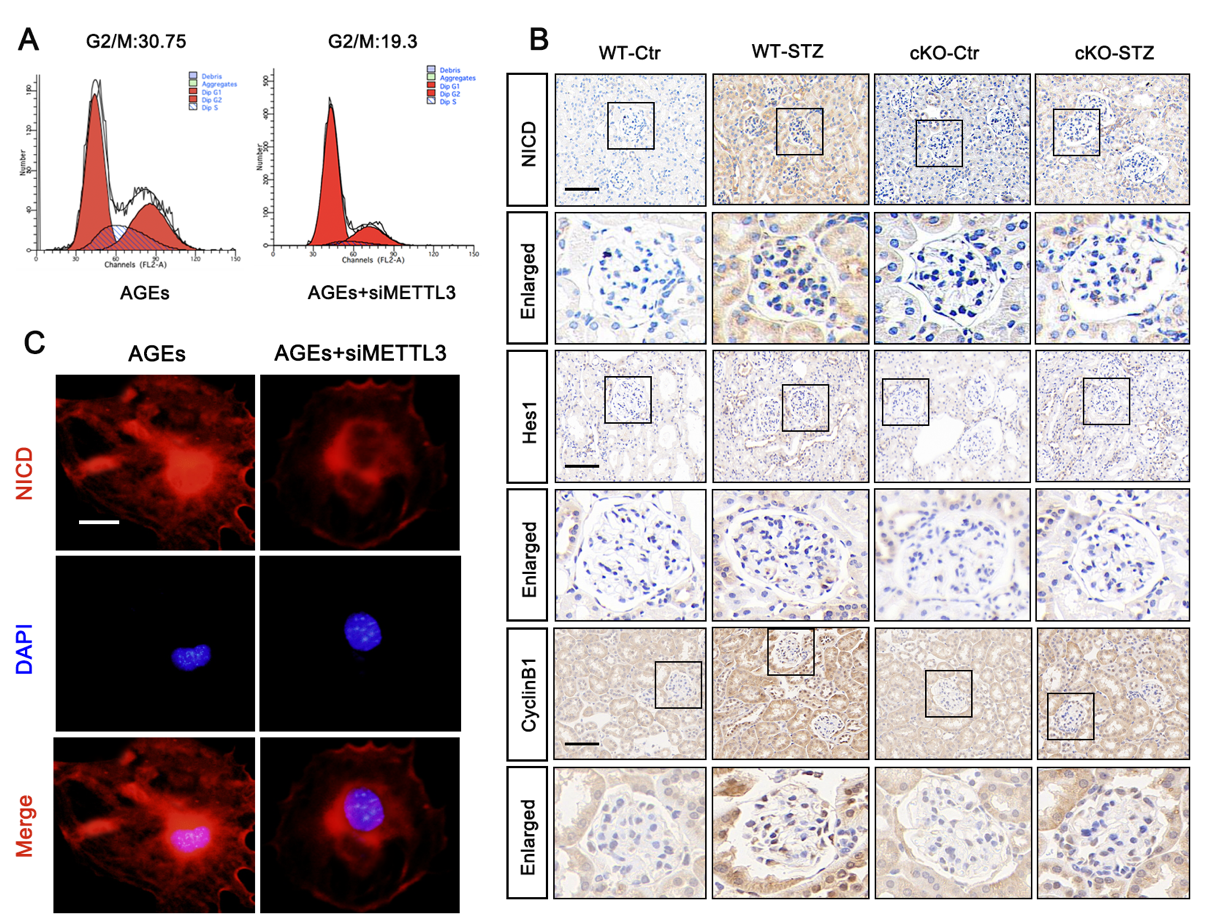


**Figure S3. METTL3 knocking out in podocytes regulates MDM2-mediated Notch1 signaling pathway and influences cell cycle stability**

(A) Flow cytometry analysis of cell cycle events in podocytes from AGE-treated and AGE-treated with siMETTL3 groups. (B) IHC assay evaluating the protein levels of NICD, Hes1, and Cyclin B1 in WT-Ctr, WT-STZ, cKO-Ctr, and cKO-STZ groups; scale bar = 50 μm. (C) Immunofluorescence staining for NICD (red) and counterstained with DAPI (blue) in AGE-treated and AGE-treated with siMETTL3 groups; scale bar = 20 μm.
